# Effect of Zirconia Nanofibers Structure Evolution on the Hardness and Young’s Modulus of Their Mats

**DOI:** 10.3390/polym13223932

**Published:** 2021-11-14

**Authors:** Vyacheslav V. Rodaev, Alexander I. Tyurin, Svetlana S. Razlivalova, Viktor V. Korenkov, Yuri I. Golovin

**Affiliations:** Institute for Nanotechnology and Nanomaterials, Derzhavin Tambov State University, Internatsionalnaya str. 33, 392000 Tambov, Russia; tyurin@tsu.tmb.ru (A.I.T.); razlivalova8@yandex.ru (S.S.R.); ya.vikkor@yandex.ru (V.V.K.); golovin@tsu.tmb.ru (Y.I.G.)

**Keywords:** zirconia nanofibers, electrospinning, microstructure, phase composition, mechanical characteristics, nanoindentation

## Abstract

Zirconia nanofiber mats containing filaments with the average diameter of less than 100 nm were fabricated. It is found that the hardness and Young’s modulus of the mats are sensitive to the microstructure, phase composition and average diameter of the zirconia nanofibers. The hardness and Young’s modulus of the prepared zirconia nanofiber mats vary from 0.86 to 1.67 MPa and from 133 to 362 MPa, respectively, wherein an increase in hardness is accompanied by the rise in Young’s modulus.

## 1. Introduction

Among all the variety of nanofibers only ceramic ones possess increased thermal and chemical resistance [1,2]. Electrospinning is a cost effective and simple method for the large scale production of ceramic nanofibers of controllable composition and diameter [1,2]. The process of ceramic nanofibers fabrication by electrospinning consists of the following three stages: preparation of the solution containing ceramic precursor and binding polymer, hybrid fibers electrospinning, drying and annealing hybrid fibers to obtain ceramic nanofibers. Nanofibers produced by electrospinning tend to form mats.

Zirconia and its precursors are widely used to obtain different advanced ceramic materials [3]. Pure zirconia exhibits three allotropic modifications: low-temperature monoclinic (m-ZrO_2_) and high-temperature tetragonal (t-ZrO_2_) and cubic (c-ZrO_2_) [4]. However, t-ZrO_2_ and c-ZrO_2_ phases can exist at room temperature if the stabilizer is introduced into pure zirconia in the required amounts. Yttria is one of metal oxides commonly used as a stabilizer. It is well known that the mechanical characteristics of bulk zirconia ceramics can be significantly improved by adding 3 mol% Y_2_O_3_ to pure zirconia to obtain tetragonal zirconia polycrystalline ceramics [4].

Zirconia nanofiber mats are ceramic nanofiber mats with a wide range of possible applications as scaffolds for solid oxide fuel cells cathodes [5,6], separators for high-power rechargeable batteries [7], filters and adsorbents [8,9,10,11], catalysts [12,13,14], gas sensors [15], bone tissue regeneration scaffolds [16], shape-memory material for artificial muscle applications [17], electromagnetic interference shielding [18], an element of a light-sensitive photodetector [19], electrode material for electrochemical devices [20], etc.

However, the functional properties of mats depend on the diameter, grain structure, phase composition and porosity of the nanofiber components. There are several ways to control the microstructure and phase composition of zirconia nanofibers via varying the calcination temperature [21], dopant concentration [22] and zirconia precursor content in the composite intermediate filaments [23].

For any zirconia nanofiber mat application the mechanical characteristics are important. Tensile testing is usually performed to obtain data on tensile stress, ultimate strain and Young’s modulus of the ceramic nanofiber mats [8,23,24]. Nanoindentation, as the preferred method for testing thin film and surface mechanical characteristics, is another approach to examining the mechanical characteristics of nanofibrous mats, which allows defining their hardness and Young’s modulus [25]. Young’s modulus values obtained by a tensile test and nanoindentation will differ due to nanofibrous mats’ anisotropy. The given mechanical characteristics are important if nanofibrous mats are under normal stress during operation, for example, acting as adsorbents, filters or catalysts in gas flows. Random orientation of the electrospun nanofibers and macroporous structure of the mats require using a spherical indenter with a curvature radius many times larger than the diameter of nanofibers and the size of macropores to obtain reliable values of hardness and Young’s modulus for nanofibrous mats [26].

The aim of this work is to study the effect of zirconia nanofibers microstructure and phase composition on the mechanical characteristics of their mats, such as hardness and Young’s modulus, using the nanoindentation method. To reveal the relationship between the morphology of zirconia nanofibers and the mechanical properties of the mats made from them is important for any practical use of the latter, and also provides prerequisites for fabricating nanofibrous materials with the required structure and functional properties.

## 2. Materials and Methods

**Figure 1:**
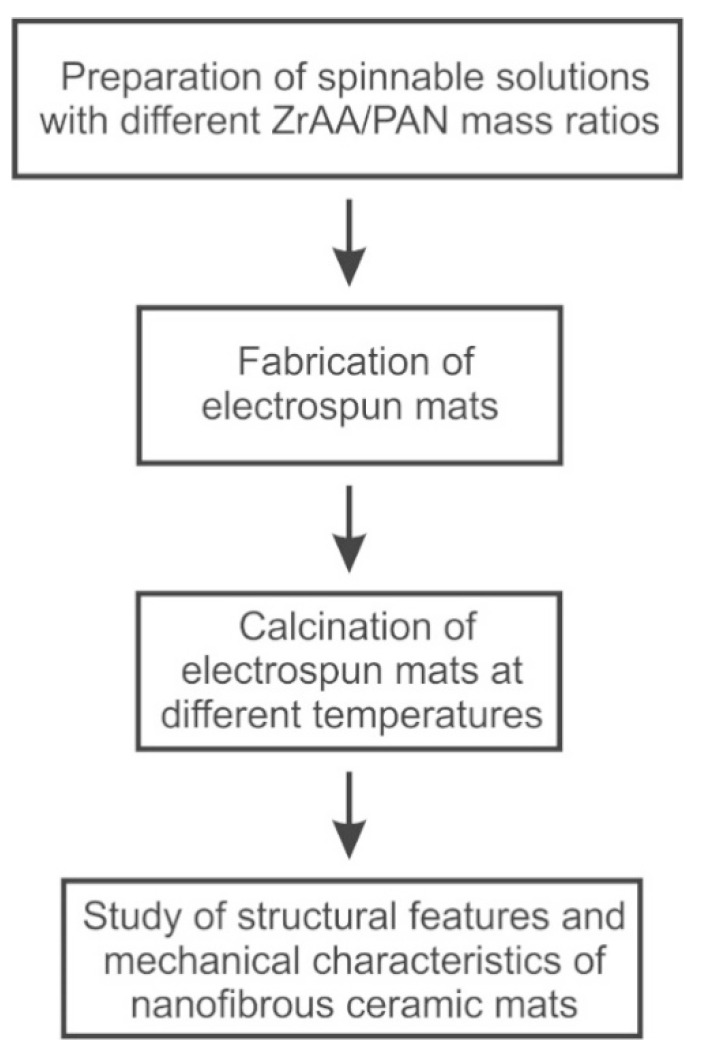
A schematic representation of the work methodology.

The mats of zirconia nanofibers with the average diameter of less than 100 nm were prepared and characterized according to the scheme shown in Figure 1.

Polyacrylonitrile (PAN, molecular weight Mw = 150,000, Sigma-Aldrich, Saint Louis, MO, USA, 1 g) was dissolved in N,N-dimethylformamide (DMF, Sigma-Aldrich, Saint Louis, MO, USA, 9 g) under magnetic stirring for 2 h at 50 °C to prepare 10 wt.% polymer solution. Zirconium acetylacetonate (ZrAA, Sigma-Aldrich, Saint Louis, MO, USA, 0.3 g) and yttrium nitrate hexahydrate (Sigma-Aldrich, Saint Louis, MO, USA, 0.015 g) were added into the prepared polymer solution and stirred at 80 °C until the solution became transparent. The amount of yttrium nitrate hexahydrate was such to obtain 3 mol% Y_2_O_3_-ZrO_2_ nanofibers. Composite solutions with ZrAA/PAN mass ratios of 0.1:1, 0.2:1 and 0.3:1 were fabricated.

The prepared composite solutions were poured into a 10 mL plastic syringe and then electrospun through a 23 G blunt tip needle upon the rectangular frame collector made of copper wire placed in a NANON-01A electrospinning machine (MECC, Fukuoka, Japan). The fibers were collected as non-woven mats. The accelerating voltage of 18 kV, the distance between the needle tip and the collector of 15 cm and a feeding rate of 1 mL/h were chosen to fabricate smooth and bead-free composite fibers.

The electrospun mats were calcined at different temperatures in air atmosphere between lightweight alumina plates with a smooth surface to prevent the mats wrinkling. Calcination was carried out in several stages in accordance with the performed thermogravimetric (TG) analysis and differential thermal (DT) analysis: heating to 320 °C with a heating rate of 1 °C/min, holding at 320 °C for 1 h, further heating to 600 °C with the same heating rate and holding at 600 °C for 1 h and finally heating to 700, 900 or 1200 °C with a heating rate of 5 °C/min and holding at the target temperature for 1 h.

The TG analysis and DT analysis were performed on the thermal analyzer EXSTAR TG/DTA7200 (SII Nano Technology, Tokyo, Japan) in air atmosphere with a heating rate of 10 °C/min.

The microstructure and diameter of the fibers were examined with a Merlin scanning electron microscope (SEM, Carl Zeiss, Oberkochen, Germany). The XRD patterns were registered in the 2θ range 20–80° by a D2 Phaser X-ray diffractometer (XRD, Bruker AXS, Karlsruhe, Germany) using CuKα1 monochromatic radiation and analyzed by means of the PDF-2 Diffraction Database File compiled by the International Centre for Diffraction. The phase content was determined from the XRD patterns by the Rietveld method using the TOPAS software (Bruker AXS) and the average grain size was also calculated in the TOPAS software (Bruker AXS) using the Scherrer equation. SEM and XRD measurements were carried out at room temperature. The specific surface area and the pore volume of the fibers were measured by nitrogen adsorption at −196 °C with a gas sorption analyzer Autosorb iQ-C (Quantachrome Instruments, Boynton Beach, FL, USA). The specific surface area was determined using Brunauer-Emmett-Teller method in a relative pressure range of 0.05–0.35. The pore volume was determined from the amount of nitrogen adsorbed at the relative pressure of 0.99.

Nanoindentation measurements were carried out by a TI-950 nanotriboindenter (Bruker AXS) at room temperature using a stabilized zirconia spherical indenter with a radius of curvature of 250 μm. A constant strain rate of 0.05 s^−1^ was kept during all the tests. The load-displacement curves were obtained under peak load of 5 mN. Peak load was held for 10 s to stabilize the possible creep in the mat. At the end of unloading a hold of 15 s was provided to correct the thermal drift. The Poisson’s ratio was about 0.25. Young’s modulus (*E*) and hardness (*H*) of the samples were calculated from the load-displacement curves using the Oliver-Pharr method [27]. The tested samples were carefully cut out from the fabricated zirconia nanofibrous mats and fixed with ethanol on the polished surface of 3 mol% Y_2_O_3_-ZrO_2_ ceramic pellets with Young’s modulus of about 220 GPa and hardness of about 14 GPa. The thickness of the tested samples was at least 50 μm.

## 3. Results and Discussion

**Figure 2:**
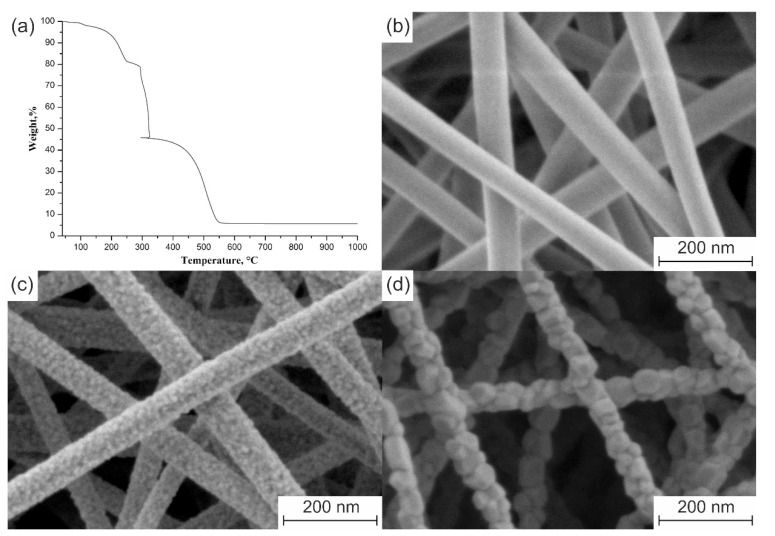
(**a**) TG curve of electrospun composite filaments with ZrAA/PAN mass ratio of 0.3:1. The microstructure of 3 mol% Y_2_O_3_–ZrO_2_ nanofibers prepared at: (**b**) 700 °C; (**c**) 900 °C; (**d**) 1100 °C from the composite filaments with ZrAA/PAN mass ratio of 0.3:1.

The 3 mol% Y_2_O_3_–ZrO_2_ nanofibers calcined at 700 °C are cylindrical in shape, with a smooth surface (Figure 2b). Their average diameter is 79 ± 7 nm. The average diameter of the filaments calcined at 900 °C is 72 ± 6 nm. No difference in the average diameters of the nanofibers calcined at 700 and 900 °C in the margin of error is associated with complete decomposition of ZrAA and PAN before 700 °C (Figure 2a).

An increase in calcination temperature from 700 to 900 °C stimulates ZrO_2_ grain growth that results in appearance of a rough nanofiber surface (Figure 2c). A calcination temperature increase to 1100 °C leads to further ZrO_2_ grains growth and also to a slight filaments shrinkage due to sintering. The average diameter of the nanofibers attains 68 ± 4 nm and their surface becomes coarse (Figure 2d). The decrease in ZrAA/PAN mass ratio in the intermediate composite fibers from 0.3:1 to 0.1:1 results in the zirconia nanofibers average diameter reduction from 72 ± 6 to 52 ± 5 nm if they are calcined at 900 °C. It seems logical that a decrease in the mass ratio of the ceramic precursor to the binder polymer leads to a decrease in the thickness of the zirconia nanofibers, since the composite filaments containing no ZrAA do not form ceramic nanofibers. It should be noted that no carbon-containing nanofibers are formed during the composite filaments’ calcination at elevated temperatures in air due to PAN burnout. To be carbonized the pre-stabilized PAN filaments must be calcined in an inert atmosphere [28].

The rise in the calcination temperature leads to the decrease in specific surface area of 3 mol% Y_2_O_3_-ZrO_2_ nanofibers (Table 1), which is associated with ZrO_2_ grain growth due to an intensification of the diffusion process. Small values of the pore volume allow classifying the prepared ceramic filaments as non-porous.

The composite filaments with various ZrAA/PAN mass ratios annealed at the same temperature produce 3 mol% Y_2_O_3_-ZrO_2_ nanofibers with a similar specific surface area.

**Figure 3:**
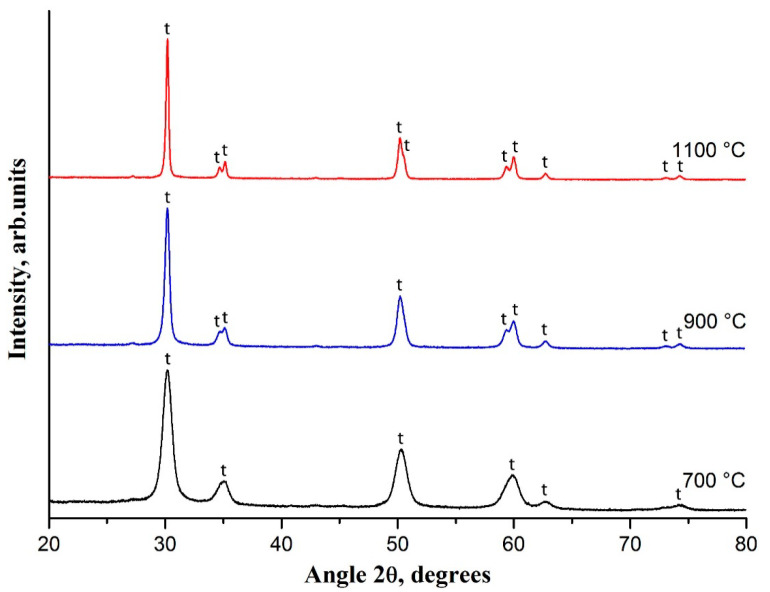
The XRD patterns of 3 mol% Y_2_O_3_-ZrO_2_ nanofibers prepared at 700, 900 and 1100 °C; t—tetragonal phase of ZrO_2_.

Figure 3 shows the XRD patterns of 3 mol% Y_2_O_3_-ZrO_2_ nanofibers calcined at temperatures in the range of 700–1100 °C. According to XRD patterns the fabricated filaments have crystalline structure and are composed purely of t-ZrO_2_ grains since the observed reflections at 30.2°, 35.2°, 50.2° and 60.2° correspond to the main peaks specific for the t-ZrO_2_ phase. With the rise in the calcination temperature the t-ZrO_2_ peaks become sharper and narrower and the reflections at 34.6°, 50.7° and 59.3° are visualized. It indicates that the crystallinity is higher and the grain size is larger for 3 mol% Y_2_O_3_–ZrO_2_ nanofibers fabricated at higher calcination temperatures. At calcination temperature of 700 °C the average t-ZrO_2_ grain size is 9 nm and it becomes 18 nm if 900 °C is used. Finally, the average t-ZrO_2_ grain size attains 31 nm at the calcination temperature of 1100 °C. SEM images confirm ZrO_2_ grain growth with an increase in the calcination temperature (Figure 2).

**Figure 4:**
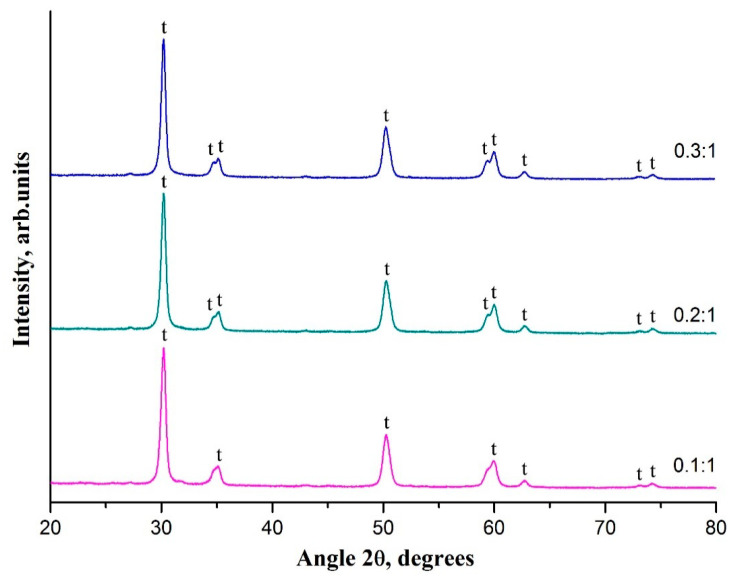
The XRD patterns of 3 mol% Y_2_O_3_-ZrO_2_ nanofibers prepared at 900 °C from the composite filaments with various ZrAA/PAN mass ratios; t—tetragonal phase of ZrO_2_.

It is revealed that the decrease in ZrAA/PAN mass ratio has no effect on the phase composition of the resulting 3 mol% Y_2_O_3_-ZrO_2_ nanofibers (Figure 4). Poorly visualized fine structure of the peaks near 35° and 60° in the XRD pattern of zirconia nanofibers prepared from composite filaments with ZrAA/PAN mass ratio of 0.1:1 may indicate a smaller size of t-ZrO_2_ grains in the nanofibers obtained from the composite filaments with lower ZrAA/PAN mass ratio. The average t-ZrO_2_ grain size in 3 mol% Y_2_O_3_-ZrO_2_ nanofibers prepared at 900 °C from composite filaments with various ZrAA/PAN mass ratios are 17.5 nm (0.1:1), 17.7 nm (0.2:1) and 18 nm (0.3:1). A slight decrease in the average t-ZrO_2_ grain size due to reduced ZrAA/PAN mass ratio may be associated with a decrease in the contact surface between the grains due to a decrease in the resulting ceramic fibers diameter that negatively affects the diffusion process. Previously, a similar effect was observed for zirconia nanofibers made of composite filaments containing zirconium oxychloride and poly(ethylene oxide) [29]. Close values of the average grain size explain the similarity in the specific surface area of 3 mol% Y_2_O_3_-ZrO_2_ nanofibers fabricated from the composite filaments with ZrAA/PAN mass ratios of 0.3:1, 0.2:1 and 0.1:1.

**Figure 5:**
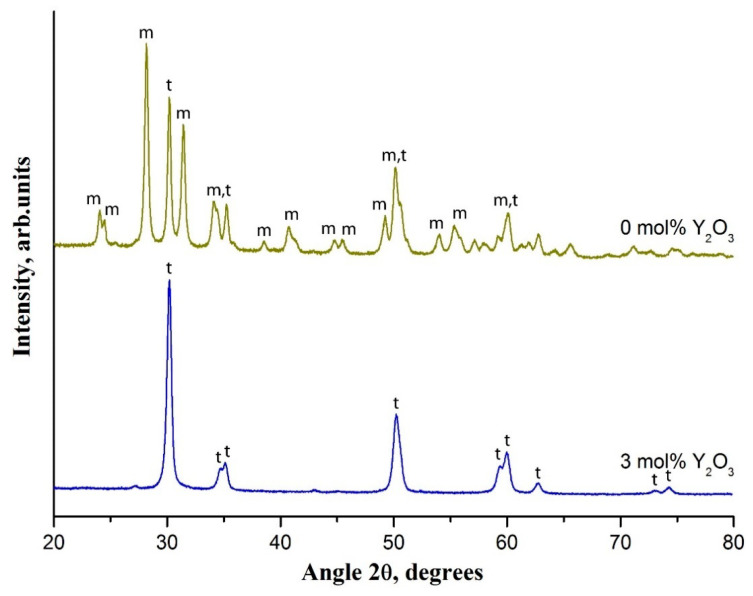
The XRD patterns of 3 mol% Y_2_O_3_-ZrO_2_ nanofibers and undoped ones prepared at 900 °C; t and m—tetragonal and monoclinic phases of ZrO_2_, respectively.

The phase composition of zirconia nanofibers changes dramatically if a dopant is not used. In the XRD pattern a large number of m-ZrO_2_ peaks appear (Figure 5).

The main characteristic peaks of m-ZrO_2_ are located at 28.2° and 31.5°. The undoped zirconia nanofibers contain 74% of m-ZrO_2_ and 26% of t-ZrO_2_ after calcination at 900 °C. In [30] it was revealed that with an increase in the calcination temperature the content of m-ZrO_2_ in undoped zirconia nanofibers rises reaching 100%. It occurs due to t-ZrO_2_ → m-ZrO_2_ transition induced by zirconia grain growth with an increase in the calcination temperature. Due to the size factor t-ZrO_2_ is thermodynamically more favorable than m-ZrO_2_ at amorphous ZrO_2_ crystallization [31]. According to Garvie’s theory the transition probability of the metastable t-ZrO_2_ phase into the m-ZrO_2_ phase increases with the rise in zirconia grain size [32]. The m-ZrO_2_ phase is stable at temperatures up to 1170 °C [33]. A dopant addition prevents this phase transition in zirconia nanofibers [34]. The t-ZrO_2_ → m-ZrO_2_ transformation is accompanied with the volume expansion due to an increase in zirconia grain size [33]. It explains the larger average grain size of undoped zirconia nanofibers prepared at 900 °C compared to 3 mol% Y_2_O_3_-ZrO_2_ nanofibers prepared at the same temperature: 24 nm and 18 nm, respectively. The larger grain size of undoped zirconia nanofibers explains their lower specific surface area compared with the 3 mol% Y_2_O_3_-ZrO_2_ ones (11.3 and 15.1 m^2^/g, respectively). This allows concluding that a small amount of yttria acts as an inhibitor of ZrO_2_ grain growth in nanofibers similarly to Y_2_O_3_ -stabilized zirconia bulk ceramics [35].

**Figure 6:**
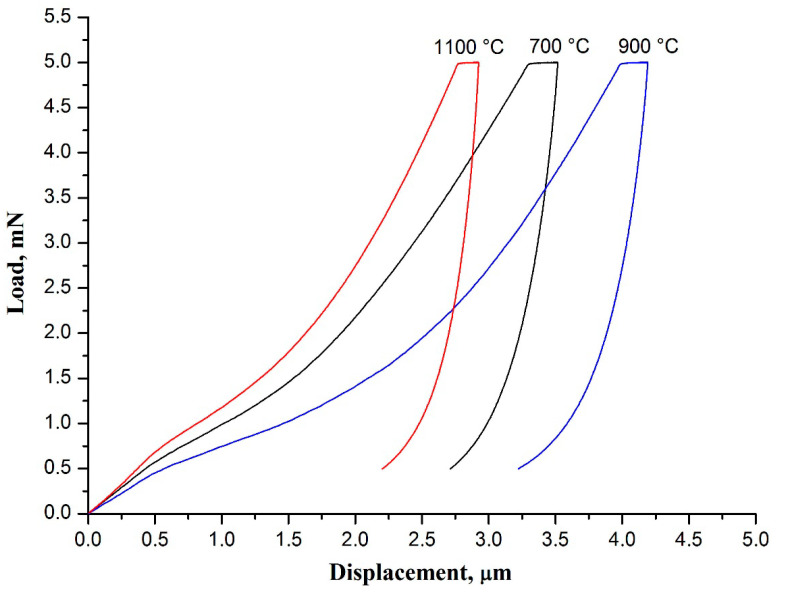
The load-displacement curves of 3 mol% Y_2_O_3_-ZrO_2_ nanofiber mats prepared at 700, 900 and 1100 °C.

Figure 6 illustrates the load-displacement curves obtained for 3 mol% Y_2_O_3_-ZrO_2_ nanofiber mats calcined at different temperatures. It can be seen from Table 2 that the mat fabricated at 1100 °C possesses the highest values of hardness and Young’s modulus.

High calcination temperature leads to formation of junctions between nanofibers at the cross-points due to sintering, which negatively affects the nanofibers freedom of movement. As a result, the mat loses flexibility and its Young’s modulus increases. Besides, the mat becomes harder and more brittle. Low calcination temperature is insufficient for nanofibers sintering at their cross-points as well as for zirconia grains sintering inside the filaments to form strong contacts. Thus, we suppose that there are no other bonds than the Van der Waals forces providing free movement of the nanofibers and the grains inside them. Therefore, zirconia nanofibrous mats calcined at low temperatures are more flexible than ones calcined at elevated temperatures. Flexible materials are known to be characterized by reduced Young’s modulus [16]. Previously, it was found that the mat of yttria-stabilized zirconia nanofibers fabricated at 1000 °C from composite filaments containing zirconium oxychloride/yttrium nitrate/poly(vinyl pyrrolidone was characterized by a lower ultimate strain in tensile testing than the one obtained at 800 °C, i.e., was more fragile [36].

The 3 mol% Y_2_O_3_-ZrO_2_ nanofibers produced at 700 and 900 °C have close average diameter values and differ in their average grain size. The smaller size provides a stronger connection between the grains due to a larger contact area, which hinders their free movement. The nanofibers become stiffer andtheir Young’s modulus increases, respectively. As a result, nanofibrous mat Young’s modulus increases too. The 3 mol% Y_2_O_3_-ZrO_2_ nanofibers prepared at 1100 °C have larger grains than filaments prepared at 700 and 900 °C. However, the connection between grains is stronger due to necks, which are formed at elevated calcination temperatures. Besides, sintering provides interfiber junctions at the cross-points as well. It explains the highest values of hardness and Young’s modulus for the 3 mol% Y_2_O_3_-ZrO_2_ nanofiber mat fabricated at 1100 °C.

Young’s modulus of the fabricated 3 mol% Y_2_O_3_-ZrO_2_ nanofiber mats is three orders of magnitude lower than that of bulk 3 mol% Y_2_O_3_-ZrO_2_ ceramics [4]. This means that zirconia nanofibrous mats are more flexible than bulk zirconia ceramics. In [16] mats of 3 mol% Y_2_O_3_-ZrO_2_ nanofibers with the average diameter of 530 ± 120 nm prepared using zirconium *n*-propoxide, yttrium acetate hexahydrate and polyvinylpyrrolidone were characterized by Young’s modulus of 1.11 ± 0.24 MPa obtained by means of microindentation. Different Young’s modulus values of the mats presented in this article and those fabricated in [16] can be associated with the different average diameter of the zirconia nanofibers forming the mats. It is shown below that Young’s modulus of the mat increases with the decrease in the average diameter of fibers.

**Figure 7:**
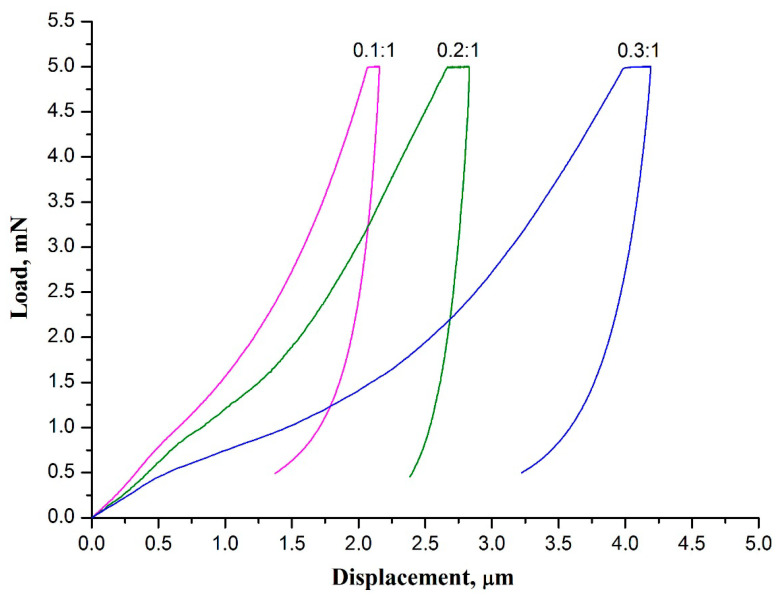
The load-displacement curves of 3 mol% Y_2_O_3_-ZrO_2_ nanofiber mats prepared at 900 °C from the mats of composite filaments with different ZrAA/PAN mass ratio.

The load-displacement curves obtained for 3 mol% Y_2_O_3_-ZrO_2_ nanofiber mats prepared at the same temperature from the mats of composite filaments with different ZrAA/PAN mass ratios are presented in Figure 7.

The 3 mol% Y_2_O_3_-ZrO_2_ nanofibers fabricated from composite filaments with different ZrAA/PAN mass ratios possess identical phase composition and consist of similar-sized zirconia grains, but differ in their average diameter, which increases with the rise in ZrAA/PAN mass ratio. A decrease in the zirconia nanofibers diameter associated with a decrease in ZrAA/PAN mass ratio results in the rise in both hardness and Young’s modulus of zirconia nanofibrous mat (Table 3). A decrease in ZrAA/PAN mass ratio produces thinner zirconia nanofibers which, in its turn, results in the rise in both hardness and Young’s modulus of a zirconia nanofibrous mat (Table 3). The higher values of hardness and Young’s modulus of the mat formed by thinner zirconia nanofibers may be explained by a reduced number of flaws. The presence of flaws in the material significantly worsens the mechanical properties of ceramics by promoting stress concentration [37]. Ceramic filaments with reduced diameter contain fewer flaws thus improving their mechanical characteristics. An increase in TiO_2_ nanofibers Young’s modulus and Al_2_O_3_ nanofibers tensile strength with a decrease in their diameter was observed in [38,39]. In [8,36] it was reported that the tensile strength of nanofibrous membranes of yttria-stabilized zirconia increases when the diameter of nanofibers decreases.

**Figure 8:**
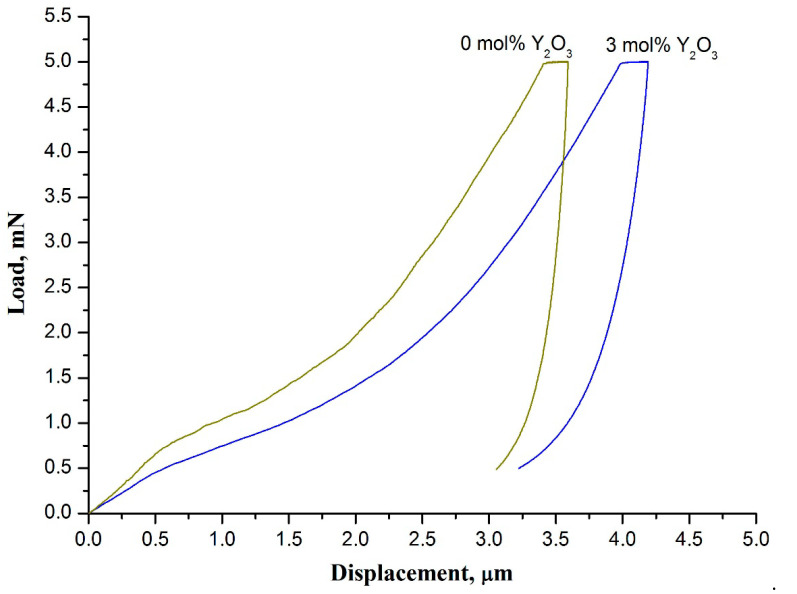
The load-displacement curves of 3 mol% Y_2_O_3_-ZrO_2_ nanofiber mat and undoped zirconia nanofiber mat prepared at 900 °C.

The results of nanoindentation measurement indicate that the hardness and Young’s modulus of zirconia nanofibrous mats are sensitive to phase composition of filaments (Figure 8).

Regarding the example of the 3 mol% Y_2_O_3_-ZrO_2_ nanofiber mat and the undoped zirconia nanofiber mat both calcined at 900 °C it is revealed that Young’s modulus and hardness of the mat of fully tetragonal zirconia nanofibers (*E* = 133 ± 4 MPa, *H =* 0.86 ± 0.05 MPa) are less than those of the mat of nanofibers mostly comprised of m-ZrO_2_ (*E* = 300 ± 10 MPa, *H =* 0.95 ± 0.04 MPa). The higher Young’s modulus and hardness of the undoped zirconia nanofiber mat indicate that it is stiffer and more brittle than the 3 mol% Y_2_O_3_-ZrO_2_ nanofiber mat prepared at the same temperature and containing no m-ZrO_2_. As mentioned above, t-ZrO_2_ → m-ZrO_2_ transition occurs if undoped zirconia nanofibers are calcined at 900 °C. Phase transition is accompanied with volume expansion which creates large internal stresses. The latter may induce nanofiber damage. It results in greater fragility of undoped zirconia nanofiber mats compared to stabilized zirconia nanofiber mats. Thus, it can be concluded that the presence of m-ZrO_2_ in zirconia nanofibers worsens the strength of both zirconia nanofibers themselves and the mats they form. An increase in the calcination temperature leads to the rise in the fragility of individual undoped zirconia nanofibers and their mats, respectively, due to m-ZrO_2_ content increase. In [30,36] it has been revealed that the mats of fully monoclinic zirconia nanofibers manufactured at 1300 °C crumble into powder upon preparation.

## 4. Conclusions

Zirconia nanofiber mats containing filaments with the average diameter of less than 100 nm were fabricated. It is found that the hardness and Young’s modulus of the mats are sensitive to the microstructure, phase composition and average diameter of the zirconia nanofibers. A decrease in the diameter of nanofibers and an increase in the heat treatment temperature leads to an increase in the hardness and Young’s modulus of the mats. The hardness and Young’s modulus of the prepared zirconia nanofiber mats vary from 0.86 to 1.67 MPa and from 133 to 362 MPa, respectively, wherein an increase in hardness is accompanied by the rise in Young’s modulus. The obtained results indicate the possibility of controlling the hardness and Young’s modulus of nonwoven zirconia membranes via varying the calcination temperature and intermediate filament composition.

## Figures and Tables

**Table 1:** The specific surface area and pore volume of 3 mol% Y_2_O_3_-ZrO_2_ nanofibers calcined at different temperatures.

Calcination Temperature, °C	Specific Surface Area, m^2^/g	Pore Volume, cm^3^/g
700	23.6	0.051
900	15.1	0.037
1100	9.3	0.022

**Table 2:** The mechanical characteristics of 3 mol% Y_2_O_3_-ZrO_2_ nanofiber mats calcined at different temperatures.

Calcination Temperature, °C	Hardness, MPa	Young’s Modulus, MPa
700	1.03 ± 0.05	171 ± 4
900	0.86 ± 0.05	133 ± 4
1100	1.25 ± 0.11	261 ± 12

**Table 3:** The mechanical characteristics of 3 mol% Y_2_O_3_-ZrO_2_ nanofiber mats prepared at 900 °C from the mats of composite filaments with different ZrAA/PAN mass ratio.

ZrAA/PAN Mass Ratio	Hardness, MPa	Young’s Modulus, MPa
0.1:1	1.67 ± 0.09	362 ± 13
0.2:1	1.11 ± 0.06	280 ± 8
0.3:1	0.86 ± 0.05	133 ± 4

## Data Availability

All data included in this study are available upon request from the corresponding author.
